# Supplementary material for: Effect of Baicalin on Wound Healing in a Mouse Model of Pressure Ulcers
Source: Int J Mol Sci. 2022 Dec 25;24(1):329. doi: 10.3390/ijms24010329 (PMC9820804; doi:10.3390/ijms24010329)
Supplement: Supplementary file 1 [file ijms-24-00329-s001.zip › ijms-2083360-supplementary.pdf]

1. Supplementary Figure

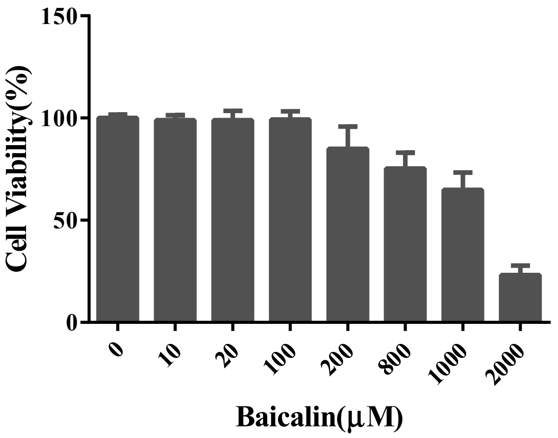

Supplementary Figure S1. Cell cytotoxicity of baicalin depending on the concentration in the Human Epidermal Keratinocytes (HEKs) cells.

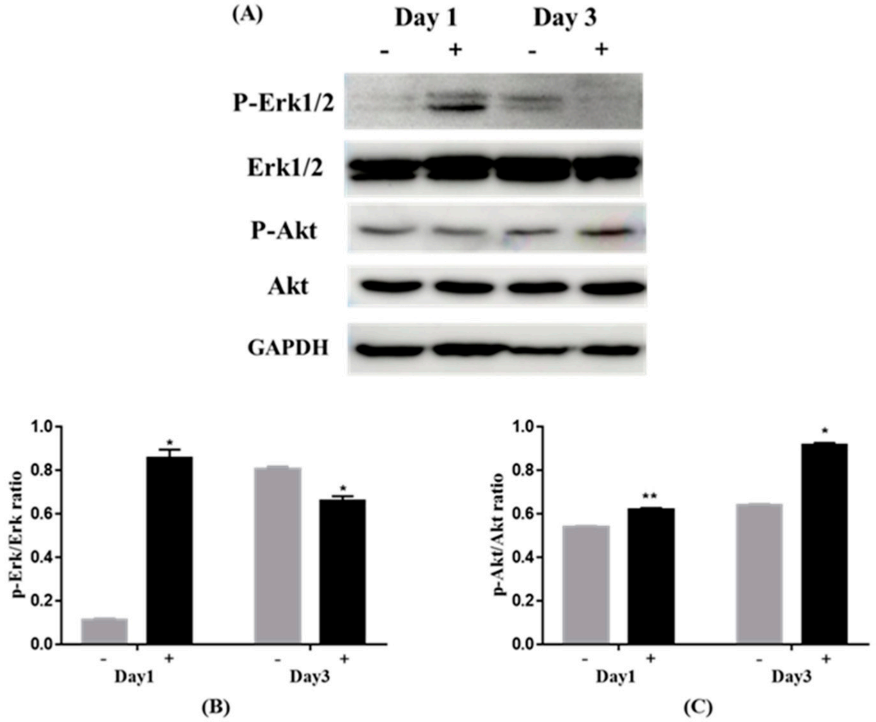

Supplementary Figure S2. Expression of proteins in pressure ulcer mouse model treated with baicalin (100 μg/mL). (A) Representative Western blot of in pressure ulcer mouse treated with baicalin on days 1 and 3 post wounding. Quantification of (B) p-ERK/ERK and (C) p-Akt/Akt expression. * $p < 0.05$, ** $p < 0.01$ phospho-extracellular signal-regulated kinase, p-Erk

2. Supplementary Table

Supplementary Table S1. qRT-PCR primers for molecular markers of pressure ulcers

**Target gene**	**Primer sequences (5′ – 3′)**
IL-1β	Forward: 5′-ACGGACCCCAAAAGATGAAG -3′<br>Reverse: 5′-TTCTCCACAGCCACAATGAG -3′
IL-6	Forward: 5′-TACCACTTCACAAGTCGGAGGC-3′<br>Reverse: 5′-CTGCAAGTGCATCATCGTTGTTC-3′
IL-10	Forward: -5′-CCATCATGCCTGGCTCAGCAC-3′<br>Reverse: 5′-TGTACTGGCCCCTGCTGATCC-3′
TGF- β	Forward: 5′-AAGGACCTGGGTTGGAAGTG-3<br>Reverse: 5′-TGGTTGTAGAGGGCAAGGAC-3′
CTGF	Forward: 5′-GGACACGAACTCATTAGAC-3′<br>Reverse: 5′-TCTCACTTTGGTGGGATAG-3′
FGF-2	Forward: 5′- GAGCGACCCTCACATCAA-3′<br>Reverse:5′- CGTTTCAGTGCCACATACC-3′
PDGF- β	Forward: 5′-AATGCTGAGCGACCACTCCATC-3′<br>Reverse: 5′-TCGGGTCATGTTCAAGTCCAGC-3′
VEGF	Forward: 5′-GCTCCGTAGTAGCCGTGGTCT-3′<br>Reverse: 5′-GGAACCCGGCGGGACACGGAC-3′
GADPH	Forward: 5′-GCACCGTCAAGGCTGAGAAC-3′<br>Reverse: 5′-TGGTGAAGACGCCAGTGGA-3′
